# 

**Published:** 1882-08

**Authors:** 


### CONTENTS:

ART. I.—The Fourteenth Annual Meeting of the Southern Dental Association......145

ART. II—Dental Society Meetings. By W. C. Barret, M. D., D. D. S..............155

ART. III.—American Medical Association Section on Oral and Dental Surgery....162

ART. IV.—Is Tobacco an Antiseptic? By Frank French, D. D. S....................168

ART. V.—Early American Dentistry. By J. B. Hodgkin, D. D. S....................172

ART. VI.—Ether vs. Chloroform. By Dr. Pedgin Teale............................176

ART. VII.—The Disorders of Primary Dentition (*Continued*). By M. Blanches......180

ART. VIII—The Decadence of the American Family..................................182

ART. IX.—Case of Interest.......................................................184

ART. X.—Cancer of Tongue.—Removal of Entire Organ. By Dr Roddick........186

ART. XI—Syphilitic Deformity of the Teeth.......................................188

### EDITORIAL, ETC.

Physical Diagnosis..............................................................189
Handpieces for Dental Engines...................................................190
Illness of Dr. M. H. Webb.......................................................190

### MONTHLY SUMMARY.

Hemorrhage and Gangrene from a Carious Tooth....................................191
The Dental Engine Modified for Surgical Use.....................................192

